# Experimental Determination of the Equivalent Moment of Inertia and Stresses of Aluminium Profiles with Thermal Breaks

**DOI:** 10.3390/ma18010023

**Published:** 2024-12-25

**Authors:** Dawid Rusin, Janusz Juraszek, Piotr Woźniczka

**Affiliations:** 1 CUT Doctoral School, Cracow University of Technology, Warszawska 24, 31-155 Cracow, Poland; 2 Aluprof S.A., Warszawska 153, 43-300 Bielsko-Biała, Poland; 3 Faculty of Materials, Civil and Environmental Engineering, University of Bielsko-Biala, Willowa 2, 43-309 Bielsko-Biała, Poland; jjuraszek@ubb.edu.pl; 4 Faculty of Civil Engineering, Cracow University of Technology, Warszawska 24, 31-155 Cracow, Poland; piotr.wozniczka@pk.edu.pl

**Keywords:** aluminium façade, FBG sensors, moment of inertia, stress, wind loads, structural monitoring

## Abstract

This paper presents the results of experimental tests and computer simulations on the stiffness of composite aluminium mullions used in unitised façades. The elements analysed were subjected to bending in order to simulate the actual operating conditions of aluminium façades subjected to significant wind pressure or suction loads. The basic mechanical and physical properties of the materials from which the analysed type of aluminium façade is made (Aluminium EN AW-6060 in the T66 temper and polyamide PA66 25GF), the test method, and the results obtained are described. As a result of the tests, equivalent moments of inertia of the composite profile (aluminium profile with the thermal break) were determined, which are strongly dependent on the strength of the connection between the individual elements, the asymmetry of the cross-section, and the properties of the thermal break. Strain measurements carried out using FBG (Fiber Bragg Grating) strain sensors installed in the profiles under tests allowed for determining the actual stress values of the aluminium profiles under consideration. The results obtained were compared to theoretical (numerical) values, indicating discrepancies at higher load values. The methodology presented in this article is to be used to monitor the deformation of the aluminium façade mullions of HRB (High-Rise Buildings).

## 1. Introduction

Façades made of aluminium are popular in the construction industry because they offer a range of advantages [1,2]. Modern design, thermal and light comfort for users and no need for maintenance (f.e. corrosion resistance [3,4]) are the most valuable of these advantages. Moreover, automated production in workshops and fast installation are important for manufacturers [5]. According to estimates (2017), 12 billion dollars were spent per year on aluminium unitized facades alone [6], which were used twice as often as stick facades (2012) [7]. In city centres in the second half of the 20th century, the number of new buildings with aluminium facades exceeded the number of buildings made of traditional masonry walls [8]. Aluminium profiles made from EN AW-6060 alloy (EN 573-3 [9]) in the T66 temper (EN 755-2 [10]) are lightweight and are characterised by high tensile strength [11]. The basic mechanical and physical properties of the alloy used are given in Table 1 [9], and its chemical composition is in Table 2.

The basic types of aluminium façades are mullion-and-transom façades and unitised façades. The first ones are characterized by the fact that most of the assembly work—such as screwing together aluminium structural elements and installing glass—takes place on the construction site. Unitised façades [12,13], on the other hand, are characterised by the possibility of transferring the burden of fabrication of the aluminium frames and their glazing to the manufacturing plant and compensating for significant expansion movements [7,11]. This has a huge impact on speeding up the work and reducing the possibility of execution errors.

An important feature of façades made of aluminium is that they can be recycled [13], making them environmentally friendly construction elements. Ease of machining and high tolerance of their manufacture allows a wide variety of shapes and structures to be created [14,15], while large glazing provides better access to natural light and contributes to thermal comfort inside the building.

Due to the fact that as much as 80% of energy in buildings is currently used for heating purposes [16], each façade should be characterized by the lowest possible thermal transmittance coefficient. This is currently 0.9 W/m^2^K [N] for window and door structures and façades [17].

In order to reduce the heat transfer coefficient and improve the thermal properties of the façade, the external and inner aluminium profiles are separated by thermal breaks, usually made of polyamide. Polyamides (PA) are a group of semi-crystalline thermoplastic polymers characterized by high chemical, thermal and mechanical resistance. PA has a low density (approx. 1.14 g/cm^3^) and a relatively low melting point (approx. 250 °C), which makes it easier to process. An important advantage of PA is its stability of shape at high temperatures, which is important in the context of sections with a thermal break due to their exposure to high temperatures during powder coating and during the actual use of the façade. To improve mechanical and thermal properties (Table 3), polyamides are often modified by the addition of glass fibre, which leads to increased stiffness, creep resistance, and dimensional stability. In construction applications, specially in façade systems, polyamides reinforced with 25% glass fibre (PA 66 GF25) are commonly used. The use of such an addition leads to a thermal expansion coefficient close to that of aluminium, which prevents the profiles from being damaged by changes in their length caused by large temperature fluctuations. The main function of PA profiles is to reduce the heat transfer coefficient between the exterior and interior of the building, which is achieved through their insulating properties.

Despite the advanced technical solutions and the aforementioned advantages of aluminium façades, it should be noted that there are some challenges in designing this type of structure. The force resulting from the action of the wind on the glass panels of the façade is transferred to the aluminium frame (made of mullions and transoms), which transmits it to the building structure via hooks and consoles [3,4]. Aluminium walls are characterised by lower rigidity compared to traditional masonry or reinforced concrete walls. Therefore, appropriate structural solutions are required. Aluminium profiles yield when exposed to wind [1,3,4,7], so sections with appropriate wall thicknesses must be used. In thin-walled aluminium profiles, tangential stresses may occur, causing excessive deformation and even loss of stability [14]. When designing aluminium façades, the requirements of the PN-EN 13830 [26] and PN-EN 1999-1-1 [27] standards should be taken into account.

In this context, this paper presents the results of our own experimental research and computer simulations concerning composite aluminium profiles. The aim of this research was to determine the actual deflections and, consequently, the actual moments of inertia of the profiles with the thermal break in the direction of wind pressure, taking into account the interaction between the individual components, i.e., the aluminium profiles and the thermal break. The obtained results were used to calibrate the FEM model intended for further parametric analyses. Deformation measurements of the aluminium profile were also carried out using FBG (Fibre Bragg Grating) sensors, which allowed the calculation of the actual stress values. The developed innovative sensor system is to be ultimately used to monitor deformations of aluminium façade mullions of HRB (High-Rise Building).

## 2. Experimental Research

### 2.1. Methodology

**Figure 1:**
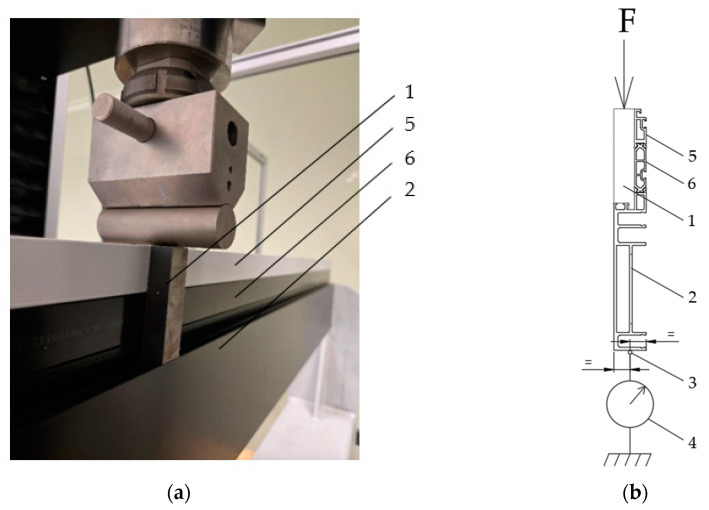
(**a**) Method of transferring the load to the profile; (**b**) diagram of the loading of the profiles by the steel cube and placement of the measuring instruments; 1—steel block, 2—inner half of the profile with a thermal break (aluminium), 3—FBG sensor, 4—mechanical displacement sensor, 5—external half of the profile with a thermal break (aluminium), 6—the thermal break profile (PA GF25).

The tests consisted of three-point bending of aluminium profiles (with and without a thermal break). The load from the cross-beam of the testing machine was transferred to the profiles using steel blocks (made of S235 steel, with dimensions of 18 × 20 × 20 mm for profiles without a thermal break and 18 × 20 × 85.7 mm for profiles with a thermal break) placed in the glazing gasket seat zone in order to reproduce the positive wind load (pressure) as accurately as possible [Figure 1].

**Figure 2:**
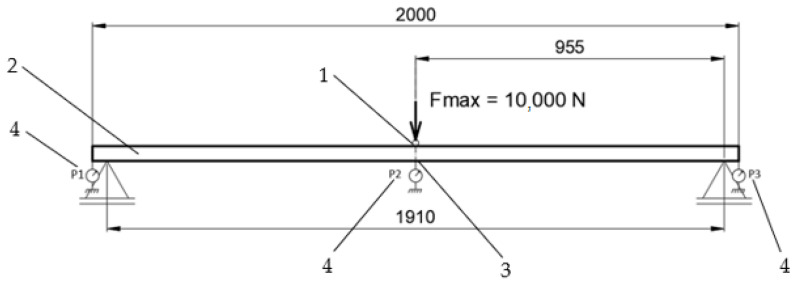
Test bench diagram; 1—steel block, 2—profile (with or without a thermal break), 3—FBG sensor, 4—mechanical displacement sensor.

The experiments were conducted on a Galdabini Quasar 50 testing machine. The machine’s load-cell reading resolution was over three million divisions (24-bit A/D converter). A load program was developed that ensures that the applied force, in steps of 1 kN, increases from 0 to 10 kN and decreases from 10 kN to 0. Displacements in the middle and two opposite ends of the tested profile (sensors P1, P2 and P3) [Figure 2], as well as deformations using the FBG sensor, were recorded. The supporting structure consisted of a steel tube (S235 JR) with a 200 × 200 × 6 mm cross-section, to which two flat bars with cut-outs (saddles) were welded, in which the tested profiles were placed. The saddles ensured vertical and horizontal stabilization of the tested profile. The spacing between supports was 1910 mm [Figure 2]. The points of measurement (P1, P2, P3) of the displacement were introduced to eliminate the effect of deformation of the supporting structure and cancel out any play in the saddles.

**Figure 3:**
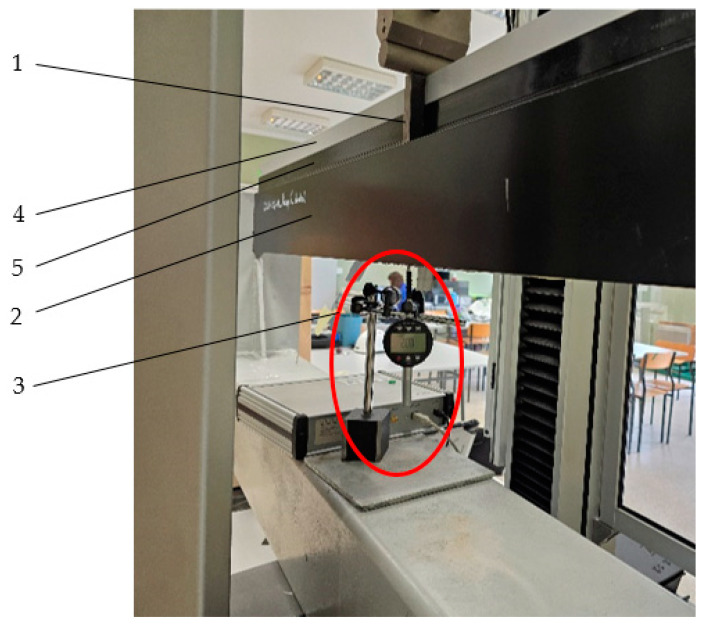
View of the mechanical displacement sensor; 1—steel block, 2—inner half of the profile with a thermal break (aluminium), 3—mechanical displacement sensor, 4—external half of the profile with a thermal break (aluminium), 5—the thermal break profile (PA GF25).

Movements were measured using mechanical dial sensors with digital displays. The resolution of the measurement was 0.01 mm, and its accuracy was 0.02 mm [Figure 3].

**Figure 4:**
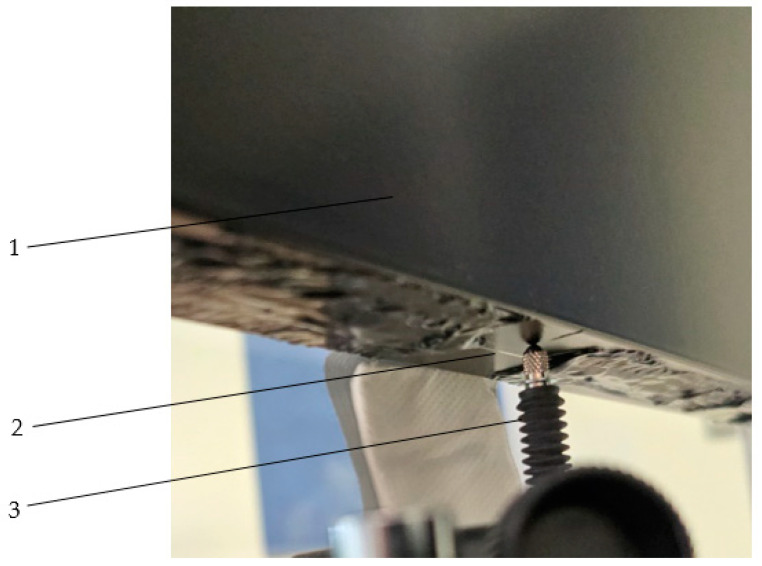
View of FBG sensor glued to aluminium; 1—inner half of the profile with a thermal break (aluminium), 2—FBG sensor, 3—mechanical displacement sensor.

FBG sensors with wavelengths from 1530–1560 nm and a maximum measurement range of 3000 microstrain (με) were used to record the strain. These were glued to the rear inner walls of the column profile halves [Figure 1b and Figure 4] using a specialized 3M DP490 Scotch-Weld epoxy adhesive.

The system was powered by light using a two-channel FOS&S FBG-scan 800 interrogator capable of operating at wavelengths of 1515–1590 nm and at a scanning frequency of 2 kHz. The ILLumiSense Strainnumer software (version v2.1.3) was used to record the strain results.

### 2.2. Subject of Tests

**Figure 5:**
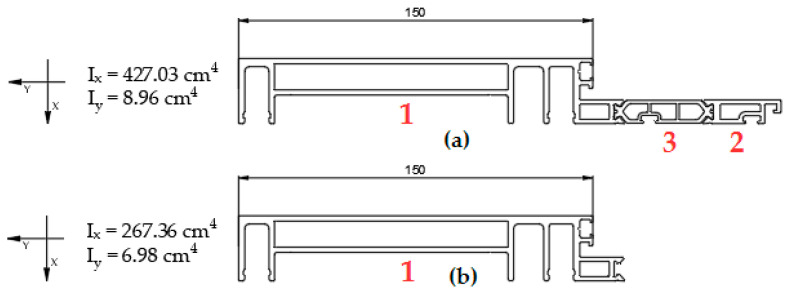
(**a**) Aluminium profile with the thermal break; (**b**) Inner half of the profile with the thermal break; 1—inner half of the profile with the thermal break (aluminium), 2—external half of the profile with the thermal break (aluminium), 3—the thermal break (PA GF25).

The mullion profiles of unitised aluminium façade with a length of 2000 mm were tested. These sections are characterized by high slenderness and cross-section asymmetry. They mainly transfer bending forces resulting from wind action. The tensile forces resulting from the weight of the glazing transferred to the mullions through the angle connectors are small, such that they can be neglected. Torsion [7] resulting from section asymmetry and the eccentricity of the load application point was neglected due to the relatively high torsional stiffness for a given profile length. The characteristic dimension of these mullions is their depth calculated from the sealing seat of the inner profiles to their rear wall. In the case of the tested pieces, it was 150 mm [Figure 5].

The tested profiles with the thermal break can be manufactured using one of two different technologies. The first of these (the so-called “single-colour”)—hereinafter referred to as technology A—involves mechanically connecting (crimping) the two profiles together using a plastic spacer and then subjecting them to a varnishing process, during which both sections are subjected to a temperature of approx. 200 °C for approx. 20 min. The profiles then go through the processes of degreasing, etching, washing, and drying [28]. The second technology (so-called “two-colour”)—hereinafter referred to as technology B—involves separating the painting of outer and inner aluminium profiles and then crimping them together without any painting processes, which could affect the plastic layer itself [28]. The first method of manufacturing is more advantageous because of logistical and economic aspects.

In addition to the profiles with the thermal break, the aluminium inner sections of these profiles were also tested [Figure 5b] for comparison purposes. Six series of tests were carried out—three for the profiles with the thermal break and three for the inner halves of the profile.

The main sources of measurement errors for this study are inaccuracies of the FBG sensors and mechanical displacement sensors and testing machine load precision. The values of these inaccuracies have been previously described.

## 3. Laboratory Results and Discussion

### 3.1. Inner Halves of Profiles with the Thermal Break

**Figure 6:**
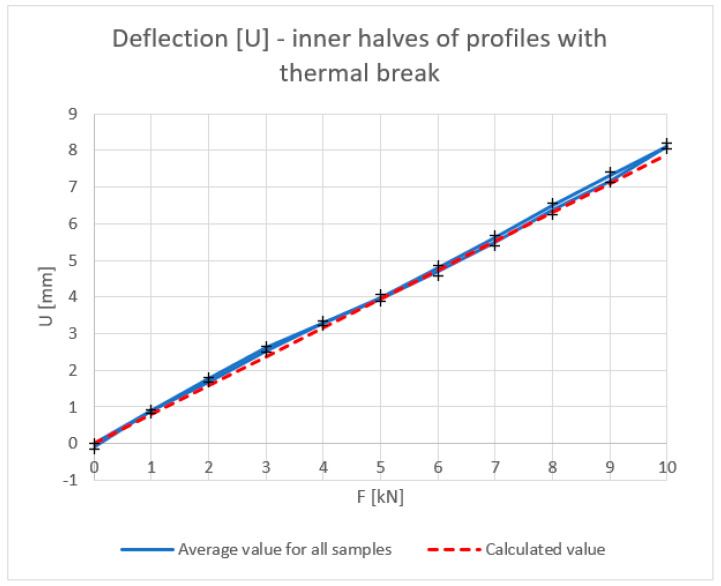
Deflection U [mm] of inner halves of profiles with the thermal break (average value for loading and unloading of the beam). Calculated value—according to the theoretical calculation.

**Figure 7:**
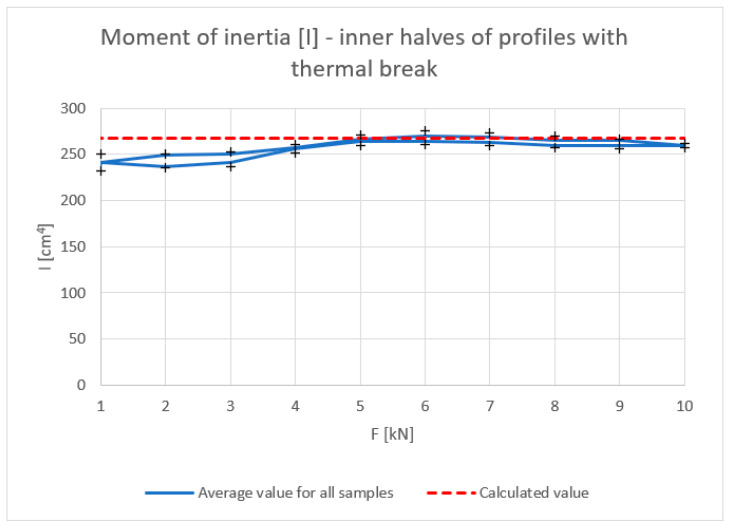
Moment of inertia I (cm^4^) of inner halves of profiles with the thermal break (average value for loading and unloading of the beam). Calculated value—according to the theoretical calculation.

**Figure 8:**
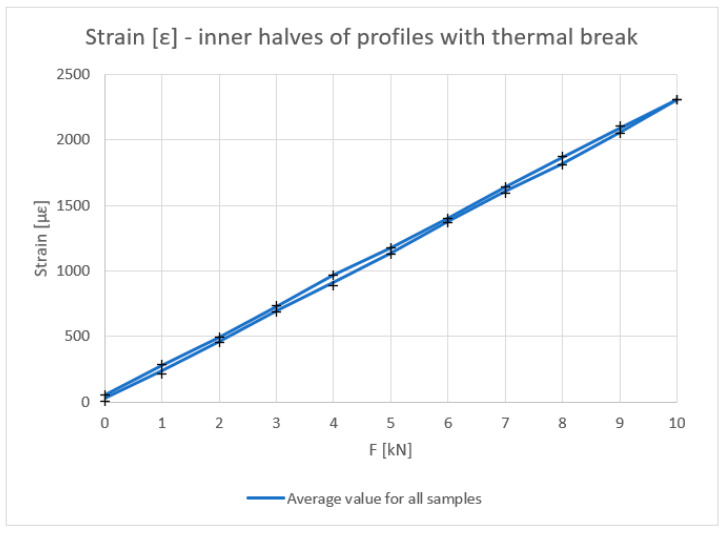
Strain ε (με) of inner halves of profiles with the thermal break (average value for loading and unloading of the beam).

**Figure 9:**
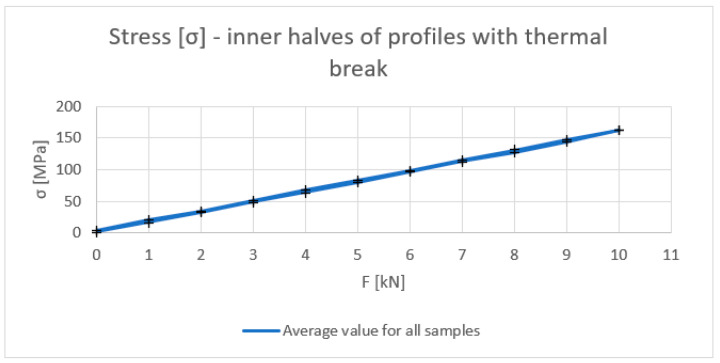
Stress σ (MPa) of inner halves of profiles with the thermal break (average value for loading and unloading of the beam).

Figure 6, Figure 7, Figure 8 and Figure 9 show the results obtained for the inner halves of the profile. In each of these, the results obtained are compared with theoretical values. The cross points in the graph are the minimum and maximum values obtained during experiments on the discussed samples.

Figure 8 shows the strain values determined based on physical relationships at the mid-length of the tested profile based on the strain measurement.

The deflection, deformation, and strain results, irrespective of the applied force, coincide with the theory, especially in the load build-up phase. A slight decrease in the mechanical parameters can be observed in the stress-relieving phase of the tested piece. The dependence of the moment of inertia on the force, especially at lower values, differs slightly from the theoretical values.

### 3.2. Profiles with the Thermal Break

**Figure 10:**
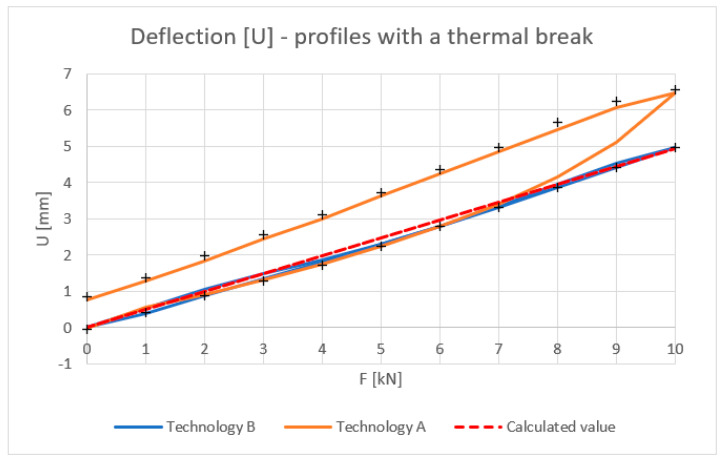
Deflection U [mm] of profiles with a thermal break (average value for loading and unloading of the beam). Calculated value—according to [29].

**Figure 11:**
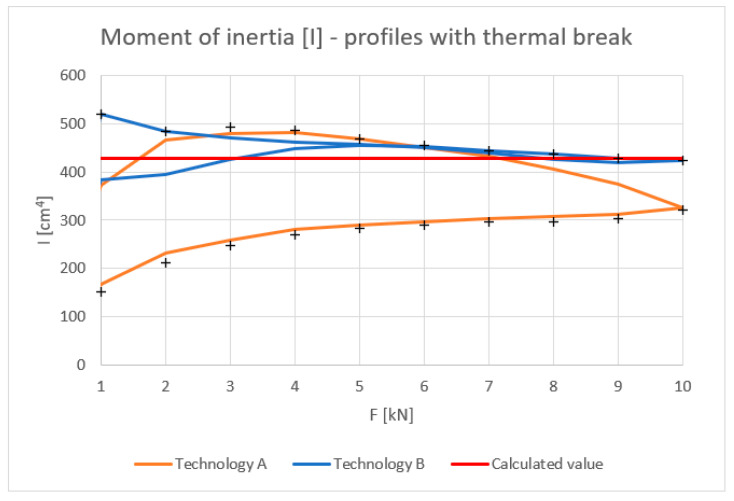
Moment of inertia I (cm^4^) of profiles with the thermal break (average value for loading and unloading of the beam). Calculated value—according to [29].

**Figure 12:**
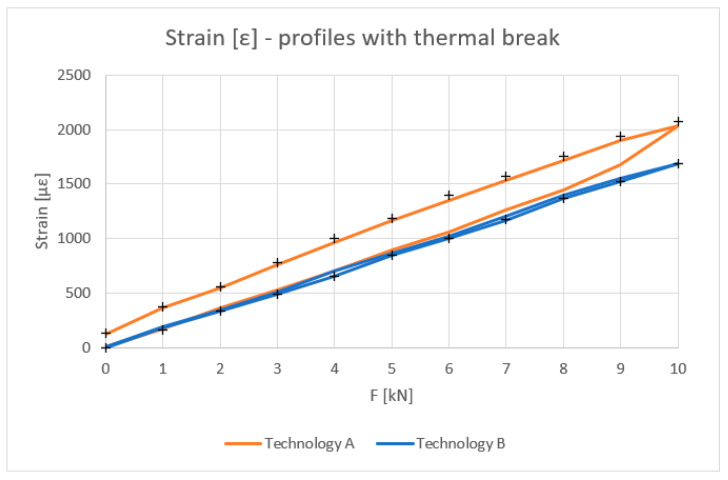
Strain ε (με) of profiles with the thermal break (average value for loading and unloading of the beam).

**Figure 13:**
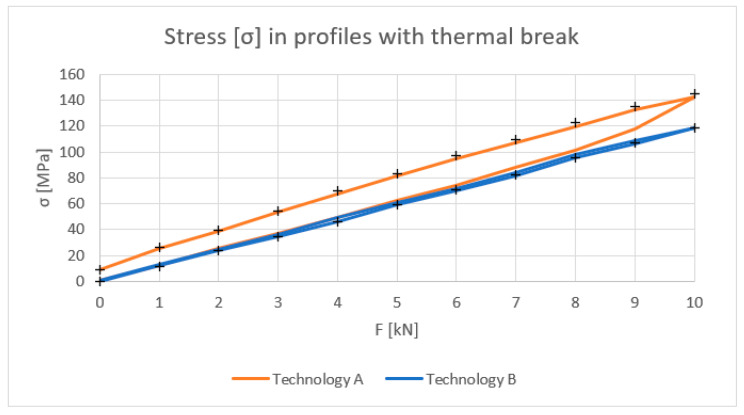
Stress σ (MPa) in profiles with the thermal break (average value for loading and unloading of the beam).

Figure 10, Figure 11, Figure 12 and Figure 13 show the results obtained for the profiles with thermal breaks. In the case of components manufactured according to single-colour technology (A), the results were presented as an average of two samples. The theoretical moment of inertia for profiles with the thermal break was calculated following the methodology given in PN-EN 14024:2004 Annex C [29]. The presented graphs show that both the deflection and deformation of the profile do not return to zero points after unloading.

Figure 12 shows the strain values determined for the profiles with the thermal break based on physical relationships at the mid-length of the profile and the deformation measurement. Values up to 8 kN bending force can be considered correct. Above this value, non-linear effects occur, probably related to the operation of the thermal break.

### 3.3. Evaluation of the Test Results

The tests showed that the results for the inner halves of the façade mullion profiles are highly consistent with theory. The average deviation of the experimental values from the theoretical values at a load of 10 kN was approximately 3%. When the load was relieved to 1 kN, the deviation averaged 9%. Profiles with the thermal break manufactured using the single-colour technology (A) at a load value of 10 kN showed a discrepancy of about 25% in relation to the theoretical values, whereas after relieving the load to a value of 1 kN, this discrepancy was, on average, 60%. The profiles manufactured in two-colour technology (B) at a maximum load of 10 kN showed a 1% discrepancy with the theory, and after their unloading to 1 kN, a deviation of about 22% was observed. The high convergence of the results with the theory in the case of the inner profiles of the profile halves is caused by the lack of additional plastic elements used as a thermal break. The beam material is homogeneous and behaves linearly in elastic range. These profiles are never used as stand-alone elements in frame structures and are always a component of profiles with thermal breaks manufactured by either A or B technology. The results of these tests confirmed that the aluminium profiles of the mullions operate within their elastic range under a given load and demonstrated the consistency of the calculated results with the obtained experimental ones. Profiles with thermal breaks that include a plastic thermal break are characterized by non-linearity. Their mechanical properties depend not only on their length, described in [29], but also on the value of the applied load, which can be seen in the result graphs. In the case of profiles manufactured using two-colour technology (A), the deviation values are the highest. As already described, their length was 2000 mm, but the distance between the points of support (saddles) was 1910 mm. According to standards [26,30], the maximum permissible deflection of curtain wall profiles between support points should not exceed d ≤ L/200 or 15 mm, whichever is smaller. For the profiles tested, it was, therefore, 9.5 mm. This value was not exceeded in any of the cases tested, i.e., for both profiles with the thermal break manufactured using single-colour technology (A) (maximum deflection of 6.5 mm) and two-colour technology (B) (maximum deflection of 4 mm). However, when analysing the nature of the decrease in mechanical parameters and the high hysteresis of profiles manufactured using technology A, it should be assumed that despite the lack of visual signs of their damage, these profiles may not guarantee their structural performances. This behaviour of the profiles can be attributed to the effect of the coating temperature on the thermal break, which is approximately 200 °C, relatively close to the melting point of its material (PA 66 GF25) of 250 °C. Additionally, the previously mentioned technological processes (degreasing, etching, rinsing, and drying [28]) performed during the painting process, if we take into account the geometry of the connection of the aluminium profiles with the thermal break, may also result in the deterioration of their mechanical properties.

The primary sources of measurement error may include inaccuracies in the load application by the Quasar 50 testing machine and imprecision in the strain measurement using mechanical displacement sensors.

**Figure 14:**
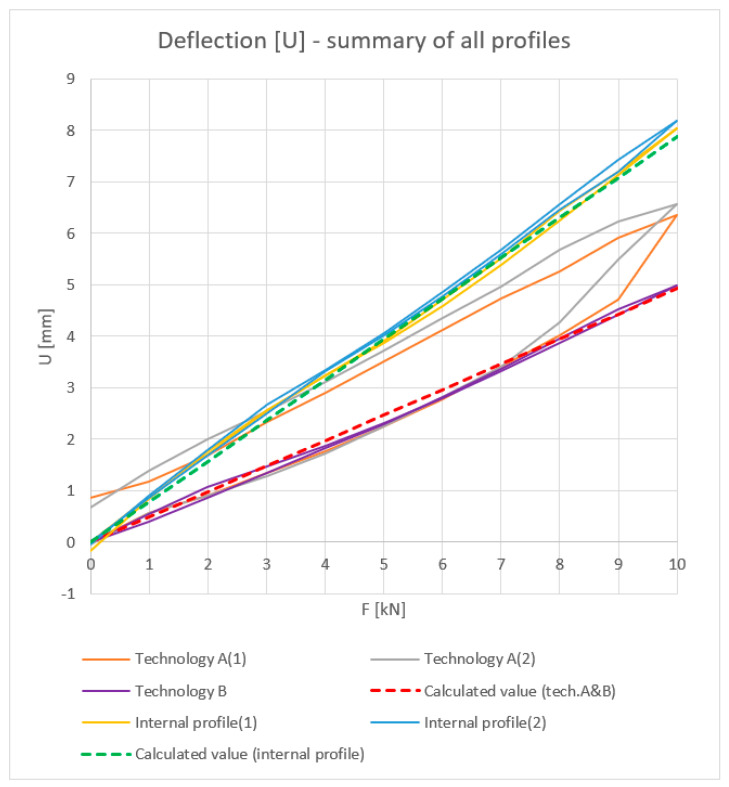
Load [kN]–Deflection U (mm): summary values of inner halves of profiles and profiles with the thermal break made in single-colour (A) and two-colour technology (B).

**Figure 15:**
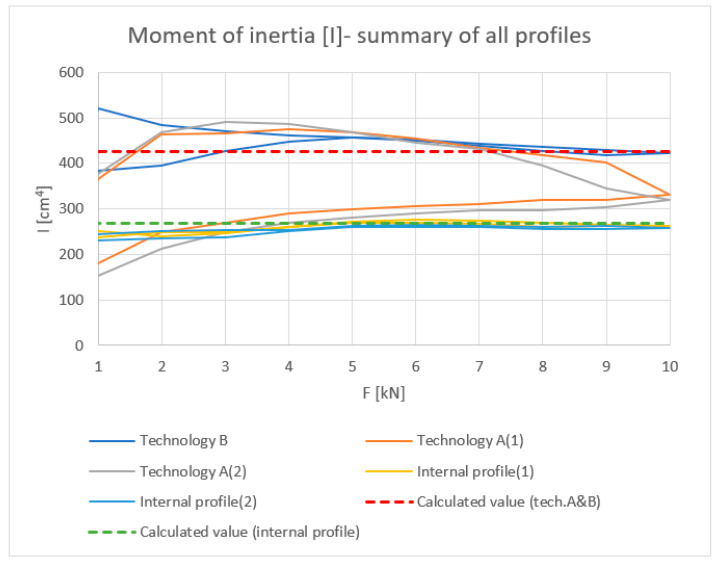
Load [kN]–Moment of inertia I (cm^4^): summary values of inner halves of profiles and profiles with the thermal break made in single-colour (A) and two-colour technology (B).

**Figure 16:**
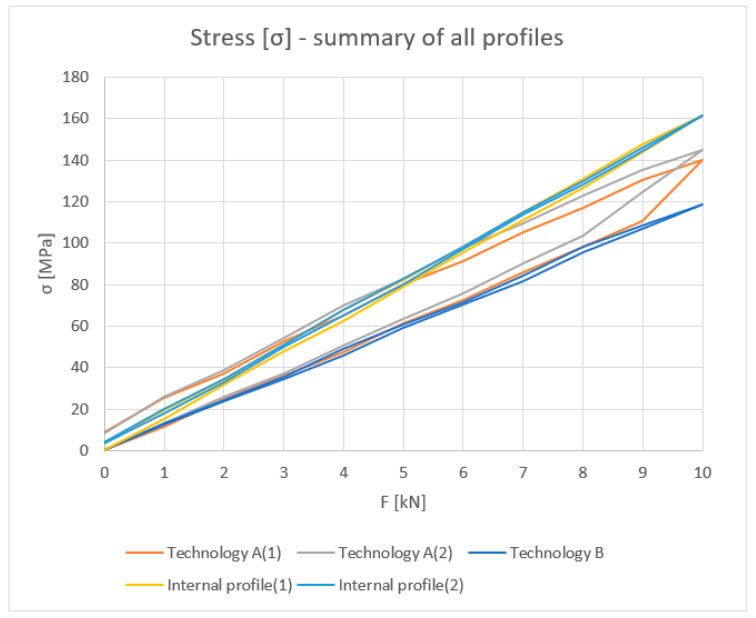
Load [kN]–Stress σ (MPa): summary values of inner halves of profiles and profiles with the thermal break made in single-colour (A) and two-colour technology (B).

**Figure 17:**
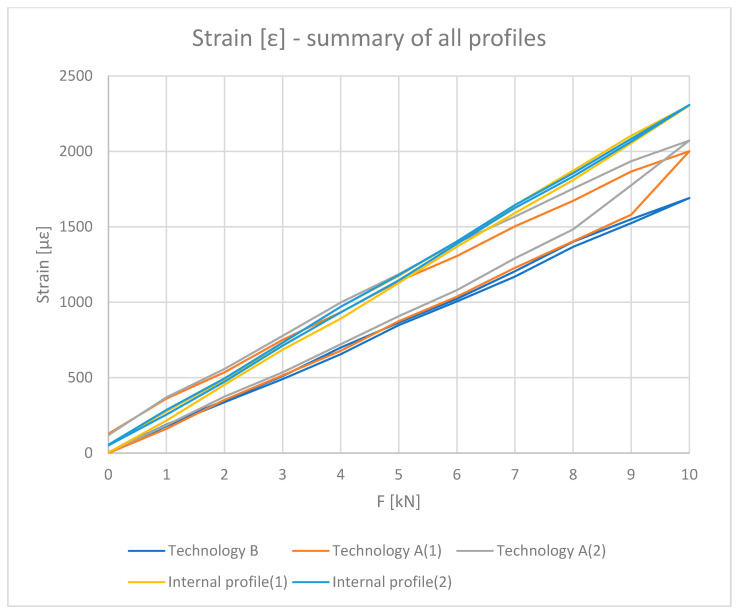
Load [kN]–Strain ε (με): summary values of inner halves of profiles and profiles with the thermal break made in single-colour (A) and two-colour technology (B).

All results are summarized in the Figure 14, Figure 15, Figure 16 and Figure 17.

### 3.4. Evaluation of the Use of FBG Sensors

During the tests, it was observed that FBG sensors perform well as devices measuring the deformation of aluminium frame profiles of unitised façades and can be implemented as part of a continuous monitoring system in HRBs. This is supported by their maintenance-free design, durability, compact dimensions, and high measurement accuracy. Their use allows the calculation of the stress occurring in the loaded profile. A major advantage is that the sensor can be calibrated to also measure profile deflections, which was verified during the tests. This process consisted of a simultaneous reading of the displacement measured using traditional displacement sensors and the deflection measured with an FBG sensor. The relationship between these values was linear; hence, by reading the deformation, the exact value of the displacement is known. The measurement method is described in more detail in the paper [31]. This makes it possible to eliminate the use of a mechanical displacement measurement system, which consists of at least three sensors (for each profile) mounted on an independent substructure, and replace it with a single optical fibre bonded to the tested element. Regarding the HRB’s continuous façade monitoring system, it is important to limit the size of the measuring and recording equipment due to the inconvenience to normal building use. In addition, FBG sensors can also record other parameters, such as the intrinsic vibrations of the profiles or their temperature. A review of the scientific literature [31,32] also confirms the validity of their selection for these purposes.

The strain measurement could be influenced by the compliance of the epoxy adhesive used to bond the FBG sensor to the profile, although this effect is difficult to quantify and is likely negligible.

## 4. Numerical Analysis and Discussion

### 4.1. Computer Model

The previously described experimental studies were used to validate the computer model developed using the Abaqus program [33]. Aluminium profiles and gaskets were modelled using general-purpose linear four-sided shell elements (S4R). The transition zones between the individual profiles, the gaskets, and the steel load element were, in turn, modelled using C3D8R solid elements (general-purpose eight-node linear brick element, with reduced integration and hourglass control). Similar approaches have previously been successfully used by the authors for computer modelling of full-scale experimental studies of a wind-loaded aluminium curtain wall [1] and also for other complex analyses of thin-walled shell structures [34,35].

The aluminium sections and gaskets were, in each case, divided into rectangular finite elements with dimensions equal to 5 × 5 mm (locally 2 to 4 mm). For the steel loading bar, a finite element mesh with dimensions of 4 × 5 × 5 mm was used. Further refinement of the basic mesh (for aluminium members, steel bar and gaskets) to a side length of 2.5 mm did not significantly affect the results. The calculated deflection values and stresses changed up to 2.5%. Taking into account computational cost, the further mesh size reduction was omitted. However, in the central part of the cross-section where the stress values were read, the mesh was densified until the convergence was reached. Finally, a local dimension of 1 × 1 mm was adopted. Simulations have been conducted on a Fujitsu Celsius M740 unit (Xeon E5-2630v3 processor; 2 × 16 GB DDR4-2133 rg ECC). Depending on the case and mesh density, the required time for a single simulation varied from just a few minutes to nearly one hour.

**Figure 18:**
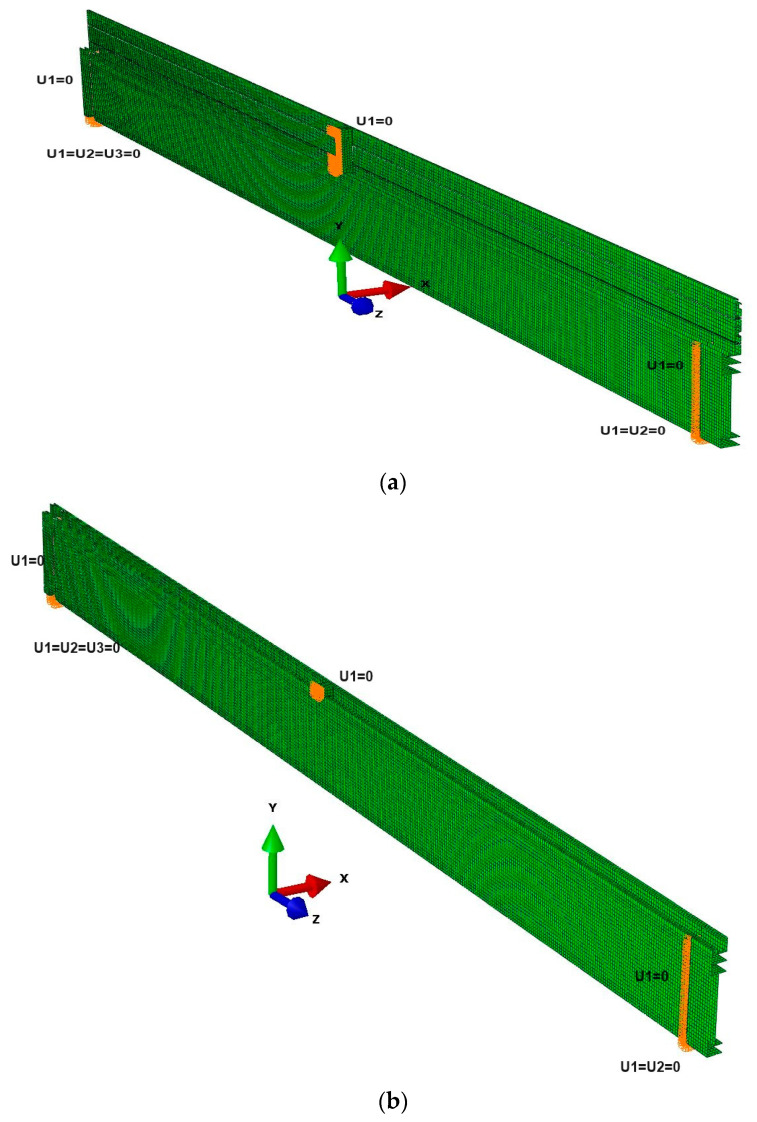
Boundary conditions of the (**a**) profile with the thermal break; (**b**) inner half of profiles with the thermal break.

Computer simulations were run as geometrically and materially non-linear (GMNA) simulations. The computational approach used, which does not take into account the influence of self-stress and geometric imperfections, can be justified by the significant slenderness of the component under test while, at the same time, the measured values of geometric deviations are negligible. For all elements, the elastic-plastic material model was used. Regarding thermal breaks made of PA 66 GF25, this approach should be considered simplistic [36] but can be justified by the expected work of the component in the elastic range resulting from the relatively low stress values. Full integration of the individual components in the computer model was assumed. The contact problem was only considered for the contact surface between the aluminium profile and the steel load bar. In the case of the aforementioned zone, a two-surface contact problem was applied. Normal (‘hard contact’) and tangential behaviour (friction) were considered. The beam load was modelled as a uniformly distributed load applied to the upper surface of the steel element. The load value was changed linearly following the assumptions made during the experimental studies. The model was supported in a fork-like manner. Additionally, a horizontal support for the steel loading element was introduced to the model. This ensured a better representation of the conditions prevailing during the tests and eliminated the numerical instabilities of the model. Views of the developed model, together with the adopted boundary conditions, are shown in Figure 18.

### 4.2. Results of the Computer Modelling

**Figure 19:**
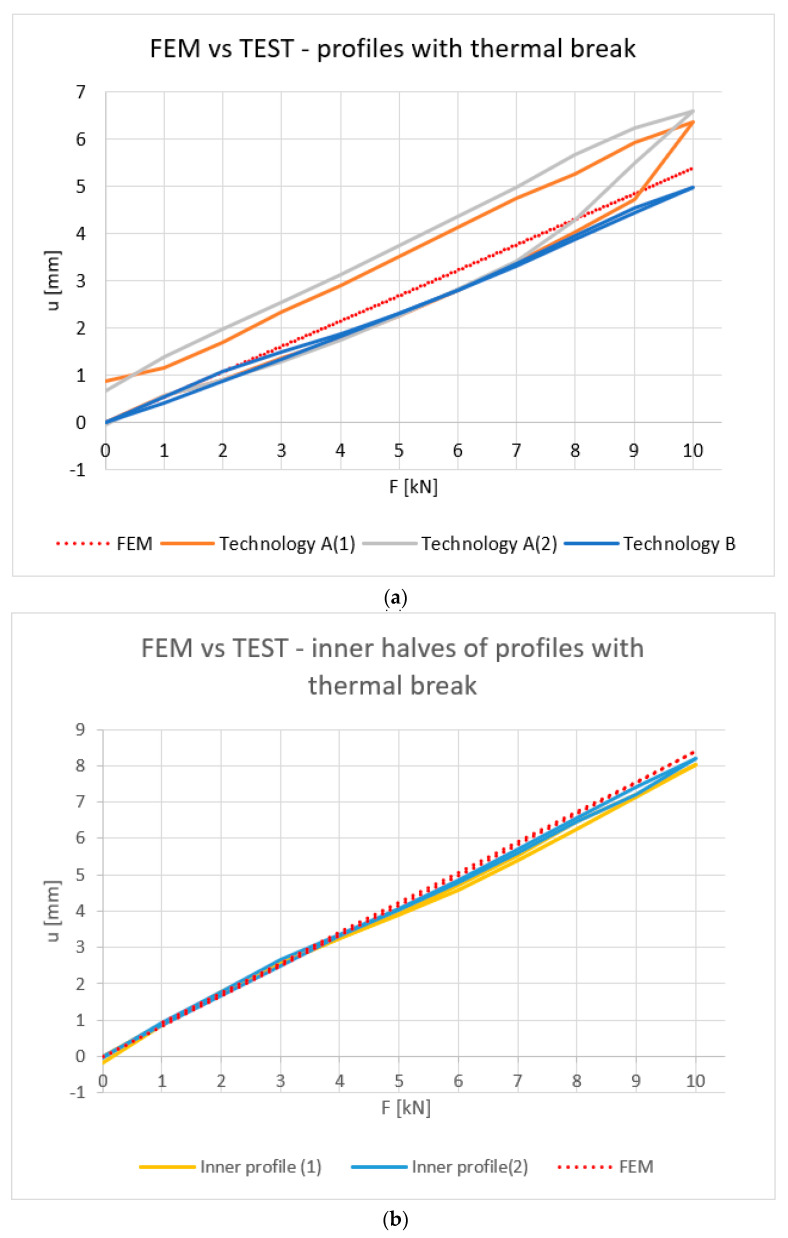
(**a**) Comparison of FEM deflection and tests—profiles with the thermal break, (**b**) Comparison of FEM deflection and tests—inner halves of profiles with the thermal break.

**Figure 20:**
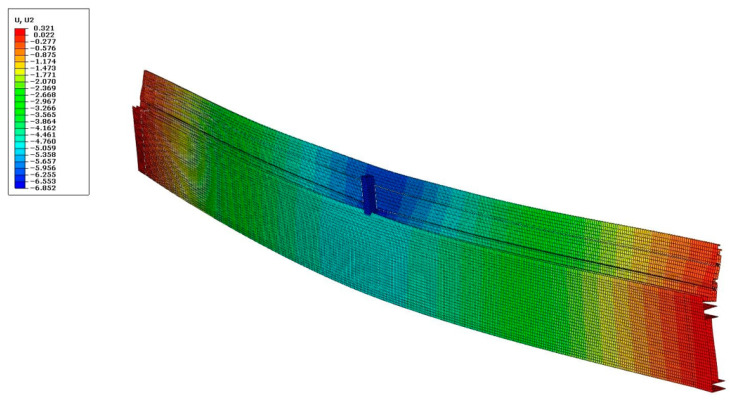
Deflection [mm] of profile with the thermal break for F = 10 kN.

Figure 19 compares the deflections obtained during the experimental tests with the results of the computer simulations. Table 4 presents the maximum deflection values determined for individual cases. Finally, Figure 20 shows the deformation of the composite profile subjected to a maximum force of 10 kN.

As can be seen from the presented data, the convergence of the results of computer modelling and experimental studies should be considered satisfactory. Maximum differences of up to approx. 20% between the FEM results and the experimental results were recorded for beams manufactured using single-colour technology A. It should be noted that in the case of models developed for these beams, the influence of heat treatment on the mechanical properties of the thermal break was not considered. However, the aforementioned treatment can lead to slight changes in the mechanical properties of aluminium, amounting to approximately 10% regarding the conventional yield strength and about 1.5% regarding the elastic modulus [37]. For the other elements, the achieved convergence of results can be considered highly satisfactory. In the case of a composite beam manufactured using the B-type (“two-colour”) technology, computer modelling led to a slight overestimation of the deflection value (maximum 8%). In contrast, for profiles without a thermal break, the modelling results led to a minimal overestimation of deflection (2% to 4%, cf. Table 4). In the case of the maximum stress values (Table 5), the differences between the computer simulation results and the experimental results were (for single-colour profiles and aluminium profiles) up to a maximum of 10%. Comparison of the stress results (Table 6) determined in the “z” direction (see Figure 18b) with the readings taken using optical fiber indicate greater discrepancies (from 12 to max. 18%). The observed differences may be caused by both the necessary simplifications of the complex geometry of the profiles and the way the seal itself works, which starts to transfer loads with a certain delay. Additionally, as already mentioned, the computer model does not take into account residual stresses, the value of which, in the case of similar aluminium alloys, can be up to 20 MPa [38,39]. Adding the mentioned value of residual stresses to the results of computer modelling leads to a safe estimation of the stress values in relation to the results of measurements performed during experimental tests. In summary, the results obtained should be considered satisfactory.

## 5. Conclusions

1. Experimental tests have confirmed the linear relationship between deformation and load for a uniform inner profile. Satisfactory convergence between theoretical, experimental, and computer simulation results has been demonstrated. For the deflections, the difference between FEM results and experimental results was up to 8% (two-colour and aluminium profiles). In relation to stresses, the final results of FEM and experimental investigations were nearly identical (difference of up to 1%).
2. Significant differences were observed in the values of deflections (up to 32%) and strains (up to 23%) for profiles with thermal breaks made using different single-colour (A) and two-colour (B) technologies.
3. The deformation hysteresis is lower in the case of profiles made using two-colour technology (B) (Figure 10), and the load–deflection curve is nearly the same for both loading and unloading cases.
4. Single-colour technology (A) is characterized by higher strain-deflection hysteresis occurring between the loading and unloading processes. It is as high as 25%. This alters the value of the equivalent moment of inertia.
5. Two-colour technology (B), in comparison to single-colour technology (A), is characterized by higher deformation stability in loading processes and a stable value of the equivalent moment of inertia.
6. Satisfactory convergence was achieved between the computer modelling results and the experimental results for the half sections and beams manufactured using two-colour technology (B). The maximum difference between FEM and experimental results was up to 8%. This should be considered acceptable. Thus, a developed computer model can be used for further parametric tests of aluminium façade elements. In the case of technology A beams, further material tests of the gaskets are necessary to determine their parameters resulting from the thermal treatment.
7. This research has shown that FBG sensors can be successfully used as continuous monitoring elements in the study of the deformation of aluminium façades.

## Figures and Tables

**Table 1:** Physical and mechanical properties of EN AW-6060 alloy for the 0–3 mm wall thickness range.

Property	Value	Unit
Alloy–Condition	AW-6060	-
Density	2.7	g/cm^3^
Elasticity modulus E	69,500	MPa
Shear modulus G	26,100	MPa
Poisson’s ratio	0.33	-
Solidification temperature	610	°C
Melting temperature	655	°C
Specific heat at 20 °C	898	J/kgK
Thermal expansion coefficient at 20 °C	23.4	µm/mK
Specific resistance	32	nWm
Thermal conductivity	209	W/mK
Electrical conductivity	54	[%IACS]
Tensile strength Rm min.	215	MPa
Tensile strength Rm max.	-	MPa
Yield point Rp02 min.	160	MPa
Yield point Rp02 max.	-	MPa
Elongation min. A50 mm	8	%
Elongation min. A	6	%

**Table 2:** Chemical composition AW-6060 alloy (data provided by the manufacturer).

Alloy Element	Amount [%]
Mg	0.35–0.60
Mn	≤0.10
Fe	0.10–0.30
Si	0.30–0.60
Si + Fe	-
Cu	≤0.10
Zn	≤0.15
Cr	≤0.05
Mn + Cr	-
Ti	≤0.10
Bi	-
Ni	-
Pb	-
Sn	-
Zr	-
Ga	-
V	-
Zr + Ti	-
Other	≤0.05
Other − total	≤0.15
Al	residual

**Table 3:** Physical and mechanical properties of the PA66 GF25 (data provided by the manufacturer).

Property	Standard	Unit	Dry Material
Melting temperature	ISO 3146 [18]	C	min. 250
Density	ISO 1183-1- or -3 [19,20]	g/cm^3^	1.3 +/− 0.05
Glass fibre content	ISO 1172 [21]	%	25 +/− 2.5
Shore D	ISO 868 [22]		82 +/− 4
Impact strength	ISO 179-1 [23]	kJ/m^2^	min. 30
Tensile strength	527-2 and -4 [24,25]	N/mm^2^	min. 80
Young’s modulus	527-2 and -4 [24,25]	N/mm^2^	4500
Elongation at rupture	527-2 and -4 [24,25]	%	min. 3

**Table 4:** Values of deflection calculated by FEM and determined during experimental tests.

	Tech. B	Tech. A (1)	Tech. A (2)	Inner Prof. (1)	Inner Prof. (2)
U_max.TEST_ [mm]	4.98	6.36	6.58	8.04	8.19
U_max.FEM_ [mm]	5.39			8.39	
FEM/TEST	1.08	0.85	0.82	1.04	1.02

**Table 5:** Maximum stress values calculated by FEM and obtained during experimental tests. RS—residual stresses of assumed value of 20 MPa [38,39].

	Tech. B	Tech. A (1)	Tech. A (2)	Inner Prof. (1)	Inner Prof. (2)
σ_max.TEST_ [MPa]	118.37	140.07	145.04	161.35	161.56
σ_max.FEM_ [MPa]	106.4			151.5	
σ_max.FEM_ + RS [MPa]	126.4			171.5	
FEM/TEST	0.90	0.76	0.73	0.94	0.94
FEM + RS/TEST	1.07	0.90	0.87	1.06	1.06

**Table 6:** Stress values in the lower strip axis calculated using FEM and determined during experimental tests. RS—residual stresses of assumed value of 20 MPa [38,39].

	Tech. B	Tech. A (1)	Tech. A (2)	Inner Prof. (1)	Inner Prof. (2)
σ_max.TEST_ [MPa]	118.37	140.07	145.04	161.35	161.56
σ_z.FEM_ [MPa]	97.0			142.2	
σ_z.FEM_ + RS [MPa]	117			162.2	
FEM/TEST	0.82	0.69	0.67	0.88	0.88
FEM + RS/TEST	0.99	0.84	0.81	1.00	1.00

## Data Availability

The original contributions presented in this study are included in the article. Further inquiries can be directed to the corresponding author.

## References

[1] Juraszek J., Woźniczka P., Rusin D. (2022). Badania eksperymentalne i symulacje komputerowe ściany osłonowej MB-SR50N HI obciążonej wiatrem. Mater. Bud..

[2] Górka M., Leśniak A. (2018). Systemy fasad aluminiowo–szklanych i ich ocena wielokryterialna. Zesz. Nauk. Politech. Częstochowskiej.

[3] Masoumeh A., Shanmuganathan G., Nima T., Benoit P.G., Hong G. (2021). Numerical study on bearing behaviour and design of aluminium sub-heads in façade systems. Thin-Walled Struct..

[4] Masoumeh A., Shanmuganathan G., Benoit P.G., Hong G. (2020). Experimental study on bearing behaviour and design of aluminium sub-heads in façade systems. Thin-Walled Struct..

[5] Coelho G.B.A., Ostapska K., Kraniotis D., Brzozovsky J., Loli A. (2024). Development of a hybrid timber and aluminium based unitized façade system resilient to the future weather conditions in Europe via monitoring campaigns and computational models. Procedia Struct. Integr..

[6] Lee A., Shepherd P., Evernden M. (2017). Optimizing the cross-sectional shapes of extruded aluminium structural members for unitized curtain wall facades. Structures.

[7] Leea A.D., Alimanza J.A., Shepherd P., Evernden M.C. (2019). Axial Rotation and Lateral Torsional Buckling of Extruded Aluminium Mullions in Curtain Wall Facades. Structures.

[8] Leea A.D., Shepherd P., Evernden M.C., Metcalfe D. (2018). Optimizing the architectural layouts and technical specifications of curtain walls to minimize use of aluminium. Structures.

[9] (2014). Aluminium i Stopy Aluminium-Skład Chemiczny i Rodzaje Wyrobów Przerobionych Plastycznie-Część 3: Skład Chemiczny i Rodzaje Wyrobów.

[10] (2016). Aluminium i Stopy Aluminium-Pręty, Rury i Kształtowniki Wyciskane-Część 2: Własności Mechaniczne.

[11] Kozicka E., Kukawska E., Rabczak K. (2019). Fasady Aluminiowe w Eurokodzie 9. Builder.

[12] Pulling L.L., Muessel D.C., Wille H.S. (1962). Wall Construction. US Patent.

[13] Al-Alimi S., Yusuf N.K., Ghaleb A.M., Lajis M.A., Shamsudin S., Zhou W., Altharan Y.M., Abdulwahab H.S., Saif Y., Didane D.H. (2024). Recycling aluminium for sustainable development: A review of different processing technologies in green manufacturing. Results Eng..

[14] Djuric Mijovic D., Cilic A., Kostic D. (2019). Lateral Torsional Buckling of Split Aluminium Mullion. Sci. J. Civ. Eng..

[15] Naqash M.T., Formisano A., De Matteis G. (2016). Aluminium framing members in Facades. Key Eng. Mater..

[16] Yılmaz E., Aykanat B., Çomak B. (2022). Environmental life cycle assessment of rockwool filled aluminium sandwich facade panels in Turkey. J. Build. Eng..

[17] (2006). Okna i Drzwi–Norma Wyrobu, Właściwości Eksploatacyjne–Część 1: Okna i Drzwi Zewnętrzne.

[18] (2022). Plastics—Determination of Melting Behaviour (Melting Temperature or Melting Range) of Semi-Crystalline Polymers by Capillary Tube and Polarizing-Microscope Methods.

[19] (2022). Plastics—Methods for Determining the Density of Non-Cellular Plastics, Part 1: Immersion Method, Liquid Pycnometer Method and Titration Method.

[20] (2022). Plastics—Methods for Determining the Density of Non-Cellular Plastics, Part 3: Gas Pyknometer Method.

[21] (2022). Textile-Glass-Reinforced Plastics—Prepregs, Moulding Compounds and Laminates—Determination of the Textile-Glass and Mineral-Filler Content Using Calcination Methods.

[22] (2022). Plastics and Ebonite—Determination of Indentation Hardness by Means of a Durometer (Shore Hardness).

[23] (2022). Plastics—Determination of Charpy Impact Properties, Part 1: Non-Instrumented Impact Test.

[24] (2022). Plastics—Determination of Tensile Properties, Part 2: Test Conditions for Moulding and Extrusion Plastics.

[25] (2022). Plastics—Determination of Tensile Properties, Part 4: Test Conditions for Isotropic and Orthotropic Fibre-Reinforced Plastic Composites.

[26] (2015). Ściany Osłonowe. Norma Wyrobu.

[27] (2004). Eurokod 9: Projektowanie Konstrukcji Aluminiowych. Cz. 1-1, Reguły Ogólne.

[28] (2017). Lakierowanie i Anodowanie–Company Presentation.

[29] (2024). Kształtowniki Metalowe z Przekładką Termiczną. Właściwości Mechaniczne. Wymagania, Sprawdzenia i Badania do Oceny.

[30] Sun L., Li C., Zhang C., Liang T., Zhao Z. (2019). The strain transfer mechanism of fiber bragg grating sensor for extra large strain monitoring. Sensors.

[31] Donghua X., Yongxiang W., Jingfeng X. (2022). Monitoring and Analysis of Building Curtain Wall Deformation Based on Optical Fiber Sensing Technology. Trans. Civ. Eng..

[32] Rusin D., Juraszek J. (2024). Innowacyjna metoda ciągłego pomiaru odkształceń ściany osłonowej aluminiowo szklanej oraz opracowanie doświadczalno-numerycznej metody analizy stanu naprężeń w konstrukcji. Przegląd Bud..

[33] Dassault Systemes Simulia I. (2010). ABAQUS: Analysis User’s Manual.

[34] Woźniczka P. (2023). Critical temperature of laterally unrestrained steel plate girders with slender section. Eng. Struct..

[35] Woźniczka P. (2024). Fire resistance and stability of non-uniformly heated steel plate girders with slender section. Thin-Walled Struct..

[36] Woźniczka P. (2022). Computer modelling of point supported laminated glass panes. Arch. Civ. Eng..

[37] Sun Y., Zhang K., Gong G. (2023). Material properties of structural aluminium alloys after exposure to fire. Structures.

[38] Zhao Y., Zhai X., Wang J. (2019). Buckling behaviors and ultimate strengths of 6082-T6 aluminium alloy columns under eccentric compression—Part I: Experiments and finite element modeling. Thin-Walled Struct..

[39] Li B., He P., Mo S., Wang J., Wang Y. (2024). Stability and design of high-strength aluminium alloy RHS members under eccentric compression. Thin-Walled Struct..

